# Pathways Into Psychosocial Adjustment in Children: Modeling the Effects of Trait Emotional Intelligence, Social-Emotional Problems, and Gender

**DOI:** 10.3389/fpsyg.2019.00507

**Published:** 2019-03-12

**Authors:** Jose A. Piqueras, Ornela Mateu-Martínez, Javier Cejudo, Juan-Carlos Pérez-González

**Affiliations:** ^1^Department of Health Psychology, Miguel Hernandez University of Elche, Elche, Spain; ^2^Department of Psychology, Faculty of Education, University of Castilla–La Mancha, Ciudad Real, Spain; ^3^Emotional Education Laboratory (EDUEMO Lab), Faculty of Education, National University of Distance Education (UNED), Madrid, Spain

**Keywords:** emotional intelligence, psychosocial adjustment, social acceptance/rejection, childhood, gender, emotional education

## Abstract

Trait Emotional intelligence (Trait EI) can be understood as a personality trait related to individual differences in recognition, processing, and the regulation of emotionally charged information. Trait EI has been considered a variable of great importance in determining psychosocial adjustment. However, most research on Trait EI has focused on adult and adolescent populations, while very few studies have explored its influence on children. The aim of this study was to analyze possible pathways into psychosocial adjustment in children by examining the combined effects of Trait EI and emotional and social problems. It also aimed to assess the possible mediating role of gender in this relationship. A total of 268 Spanish children participated in this study, ranging in age from 8 to 12 years (mean age = 10.09, *SD* = 1.32, 45.10% male). Selected measures were applied through a web-based survey called DetectaWeb. The regression and mediation/moderation analyses confirmed that psychosocial adjustment in children was determined by Trait EI directly and by emotional and social problems in an indirect way. Together, the three variables explained 46% of the variance in psychosocial adjustment, although Trait EI was the most powerful predictor (44%), demonstrating incremental validity over and above social and emotional problems. In addition, gender was shown to be a moderating variable between Trait EI and psychosocial adjustment; for girls specifically, lower Trait EI scores were a determinant of lower levels of psychosocial adjustment, regardless of emotional and social problems. It can be concluded that the identified pathways provide keys for emotional education interventions aimed at promoting psychosocial adjustment, well-being, and good mental health among children. Our findings support the buffer role of Trait EI against maladjustment risk in children, but more clearly in girls.

## Introduction

According to the World Health Organization [WHO] (1948) classical definition, health is understood as a state of complete physical, mental, and social well-being, and not just the absence of disease or infirmity. This definition includes such diverse aspects as physical health, mental health, well-being, and psychosocial adjustment and is consistent with the biopsychosocial model that includes biological, psychological, and social factors to understand health and illness (Engel, 1977).

Psychosocial adjustment or adaptation refers to people’s capacity to adapt to the environment, which implies that the individual has sufficient mechanisms to feel good, integrate, respond adequately to the demands of the environment, and achieve his or her objectives (Madariaga et al., 2014). In childhood, psychosocial adjustment often refers to adaptation and functioning in some of the main areas that characterize this stage: family and school settings.

Among the determinants of child psychosocial adjustment, the role of emotional intelligence (EI) as a protective or promoting factor and the role of emotional and social problems as risk factors or negatively associated factors have been investigated.

Despite varying definitions of EI, however, the general consensus is that the construct is useful for identifying the necessary skills for understanding and regulating emotions, which could direct behavior and thoughts and enhance performance (Mikolajczak et al., 2015; Santos et al., 2018).

### Trait Emotional Intelligence and Health

A large meta–analysis by Martins et al. (2010; see also Petrides et al., 2007) established that EI is a positive predictor of mental health, especially if it is measured through particular self-reports instruments such as the Trait Emotional Intelligence Questionnaire (TEIQue). Extensive available evidence suggests EI is a buffer of stressful circumstances for mental health in adolescents and adults (e.g., Petrides et al., 2017; Davis, 2018).

According to the Keefer et al. (2018) explanation of the pathways from EI to health, high-EI individuals: (a) Have a healthier emotional and physiological stress response; (b) Are less likely to avoid, ignore, or distract themselves from the stressful situation as the primary way of coping, and more likely to deal with the stressor directly, thereby shortening the duration of the stressful experience; (c) Are less likely to passively dwell on the stressful situation or numb their feelings with substances, which might compound the health risks; and (d) They use more constructive ways of coping with health threats and chronic illnesses. Nevertheless, although this relationship between EI and health is well established for young people and adults already, it has hardly been replicated in children until now.

An overcome controversy in conceptualizing the construct of EI has given rise to the proliferation of various theoretical models (Vesely Maillefer et al., 2018). Despite this ongoing debate, two main research streams are recognized in the field of EI, namely, EI as a personality trait (i.e., Trait Emotional Intelligence or TEI) and EI as a cognitive ability (i.e., Ability EI or AEI) (Barchard et al., 2016). Specifically, this study is based on one of the most scientifically supported models currently available in both educational and clinical settings, the TEI theory proposed by Petrides and Furnham (2001) and reviewed by Petrides et al. (2016). According to this model of EI, TEI is a constellation of affective self-perceptions (i.e., trait emotional self-efficacy) and dispositions which facilitate emotional competence in everyday life (e.g., Kotsou et al., 2011; Mikolajczak et al., 2015); it is considered to provide a more comprehensive operationalization of the affect-related aspects of personality than the general Big Five models (Pérez-González and Sanchez-Ruiz, 2014; Vernon et al., 2008). In short, TEI can be understood as a personality trait that captures individual differences in recognition, processing, and the regulation of emotionally charged information, with generally adaptive effects for social efficiency and emotional well-being (e.g., Pérez-González and Sanchez-Ruiz, 2014; Petrides et al., 2016; Lea et al., 2018).

The study of TEI has aroused great interest in the scientific community over the last two decades for two main reasons. The first is that TEI has been associated with higher levels of psychosocial adjustment, which has been interpreted in very different ways (as general adaptation levels, psychological well-being, mental and physical health, emotional adjustment, and life satisfaction) through both correlational and experimental studies in both non-clinical and clinical samples (e.g., Martins et al., 2010; Andrei and Petrides, 2013; Costa et al., 2014; Laborde et al., 2014; Resurrección et al., 2014; Barchard et al., 2016; Petrides et al., 2017; Lea et al., 2018). A rich line of these investigations is based on the assessment of TEI through the TEIQue forms, which is directly based on the TEI theory (Petrides et al., 2016). The second reason is that research has demonstrated that TEI is not totally fixed after adolescence, since it has been shown to be modifiable through psychoeducational intervention (i.e., emotional education), with an average improvement about 15%, which is also reflected in biological changes, such as a 14% drop in diurnal cortisol secretion in Kotsou et al. (2011) study and a 9.7% drop in glycated hemoglobin in Karahan and Yalcin (2009) study, as summarized by recent comprehensive reviews of the literature (Pérez-González and Qualter, 2018) and meta-analyses (Hodzic et al., 2018; Mattingly and Kraiger, 2019).

It is necessary to emphasize that so far the TEIQue for Children, namely Child Full and Short Forms (TEIQue-CF and TEIQue-CSF, respectively), is the only EI measure based on a sampling domain that has been specifically developed for children, rather than on an expedient adaptation of the adult sampling domain, which would have been unsuitable for catching the development characteristics of children (Mavroveli et al., 2008; Banjac et al., 2016).

Studies using children samples have generally found a positive relationship between TEI and a variety of psychosocial adjustment markers, such as, higher academic achievement, better peer relations and social competence, such as nominations from peers and teachers for positive social attributes, like leadership and kindness (Petrides et al., 2006; Mavroveli et al., 2008; Agnoli et al., 2012; Andrei et al., 2015; Banjac et al., 2016). Likewise, these studies have also demonstrated a risk profile in children with low levels of TEI, expressed in the form of psychopathology, anxiety, special education needs, or truancy rates (e.g., Banjac et al., 2016; Petrides et al., 2016; Stassart et al., 2017).

Some studies have supported the utility of TEI (assessed via TEIQue) in the prediction of criteria related to health and socioemotional well-being across samples of adults, adolescents, and children (Andrei et al., 2014). Despite this, there has been little consolidated research carried out on the combined effects of TEI and social-emotional problems on psychosocial adjustment specifically in populations of children. Particularly, there is little available research that has explored the mediating effect of gender on the relationships between TEI and child psychosocial adjustment.

### Social and Emotional Problems

One determining aspect of child psychosocial adjustment and complete well-being, both present and future, involves peer relationships (Mavroveli et al., 2009; Inglés et al., 2010; Domitrovich et al., 2017). Being socially accepted and having friends is associated with good adaptation, personal well-being, increased school performance, high self-esteem, and a positive and pleasant feeling within the group (Wentzel, 2003).

However, not all children enjoy positive social interactions with their peers; peer rejection situations remain a reality in most classrooms and are considered a risk condition for developing psychosocial adjustment problems in childhood, as in later stages of life. Students involved in rejection situations often show a less adaptive psychosocial profile, in terms of life satisfaction, as well as depressive symptomatology, among other aspects of maladaptation. In addition, those children who fail to develop adequate emotional competence will find it more difficult to adapt and will continue to be more vulnerable to social rejection (e.g., Denham et al., 2003; Buck, 2014).

Concerning emotional problems, the consequences of suffering from emotional problems, such as anxiety and depression, are well documented as correlates of low levels of psychosocial functioning and adjustment. Consequently, some consequences associated with a sad, angry, and/or anxious mood are also associated with low self-esteem and self-confidence; personal, social, and school imbalances; and a higher probability of presenting externalizing problems and disruptive behaviors (Aluja and Blanch, 2004; Park et al., 2010; Reinke et al., 2012).

### Relationship Between TEI and Emotional and Social Problems

Although good emotional functioning and problems in emotional and social functioning have been analyzed as determinants of psychosocial adjustment, few studies have attempted to analyze the relationships of mediation and moderation between these variables when predicting psychosocial adjustment in children. In particular, the specific relationship between the TEI and emotional and social problems has been analyzed.

Thus, some research has highlighted the influence of EI on social relations, given its role in enabling empathy and bonding with other people. In the adolescent stage, students who are more skilled in identifying the feelings of others can use this information to show empathy and/or regulate their own emotions, as well as finding ways to better adapt their behavior to social situations, which can improve social acceptance and lead to more satisfactory relationships with peers (Palomera et al., 2012; Mancini et al., 2017).

Accordingly, children with greater emotional awareness, one of the main constituents of EI, tend to be more popular among their peers, and show more empathic and pro-social behaviors, as well as experience more positive social relationships (Garner, 2010; Von Salisch et al., 2014; Finlon et al., 2015). An association has also been established between a deficit in emotional awareness and social and personal maladjustment, school problems, and aggressiveness (Veirman et al., 2011). EI plays an important role in emotional problems, given that a high EI is associated with less social stress, anxiety, depression, and clinical imbalance in young people (e.g., Barraca and Fernández, 2006).

### Gender Differences in TEI, Social and Emotional Problems, and Psychosocial Adjustment

The scientific literature has revealed the existence of gender differences in TEI, social and emotional problems, and psychosocial adjustment, as well as in the relationship between them. Regarding gender differences in EI, research shows that the degree of precision in the emotional perception of others is an indicator of adjustment, especially in boys (Palomera et al., 2012). Girls, on the other hand, usually perceive and recognize emotional expressions better than boys (Boyatzis et al., 1993).

In relation to emotional problems in boys, most studies agree that girls score higher in the symptoms of internalized disorders (anxiety and depression, in the form of dissatisfaction with themselves, shyness, and greater feelings of sadness, anguish, or shame) (e.g., García-Olcina et al., 2014; Del Barrio and Carrasco, 2016; Losada et al., 2017). With regard to psychosocial adjustment, studies indicate a slight tendency for girls to show more personal maladjustment. In addition, according to Nolen-Hoeksema and Girgus (1994), after adolescence, girls are twice as likely to suffer from certain emotional problems, including depression and reduced self-esteem, self-efficacy, and satisfaction with life. Furthermore, some studies have showed that gender moderates the association of TEI with actual social status for early adolescents, and with perceived social status for children. Therefore, TEI predicts perceived social acceptance only for female children and adolescent (Andrei et al., 2015). On the other hand, boys present more problems of adjustment at the social level and more problems of an externalizing nature (reflected in a lack of discipline and aggressive behavior) (e.g., Keiley et al., 2010). Nevertheless, this issue is still controversial because some studies show that child maladjustment is not related to age or gender (Chen et al., 2014).

### The Present Study

There is no doubt that psychosocial adjustment is a complex phenomenon, given the diverse range of variables involved. This study aims to provide evidence of the direct influence of the TEI on child psychosocial adjustment, and its indirect (mediational) influence through indicators of emotional (emotional problems) and social (acceptance/social rejection) adjustment. In addition, this study will examine whether gender has a moderating effect on the relationship between TEI and the level of psychosocial adjustment in a sample of Spanish children.

Based on our previous review of the literature and according to our rationale, we advanced the following hypotheses: (1) TEI will be significantly and directly associated with psychosocial adjustment; (2) TEI will explain part of psychosocial adjustment through its positive relationship with emotional and social adjustment; (3) The direct and indirect relationship between the TEI and psychosocial adjustment will be moderated by gender.

In this way, this study will provide evidences to identify key elements to support the implementation of actions to prevent psychosocial maladjustment, through the early identification and intervention programs in schools and homes.

## Materials and Methods

A total of 268 boys and girls aged 8–12 participated in this study (*M* = 10.09 years, *SD* = 1.32). The children were students in the 3rd, 4th, 5th, and 6th grades of Primary Education, attending two public educational centers in the Province of Alicante (Spain). Of the participants, 121 (45.10%) were male; the sample showed a mostly medium socioeconomic level (*n* = 57, 21.3% low; *n* = 123, 46.6% medium; and *n* = 84, 31.3% high). No significant relationship of interdependence between gender and age probability distributions was found (χ^2^ = 9.89, *p* = 0.08; Cramer’s *V* = 0.19, *p* = 0.08). There were likewise no significant relationships between gender and socioeconomic level (χ^2^ = 1.74, *p* = 0.42; Cramer’s *V* = 0.08; *p* = 0.42) or age and socioeconomic level (χ^2^ = 12.49, *p* = 0.25; Cramer’s *V* = 0.22; *p* = 0.25).

### Assessment Instruments

#### Sociodemographic Characteristics

– Age, gender, nationality, and descriptive information about socio-economic status (SES) were collected. Specifically, we administered *ad hoc* items for age and gender, and a specific SES measure, the Family Affluence Scale (FAS).
– Family Affluence Scale (FAS; Currie et al., 1997). This instrument is a measure of socioeconomic level. The FAS assesses household purchasing power or family wealth and consists of four items relating to family car ownership, having one’s own (unshared) room, the number of computers at home, and the number of times the child has been on vacation during the past year. The FAS is scored in categories ranging from 0 to 7: low (0–3), intermediate (4–5), and high (6–7). The scale was developed to reliably estimate family SES in (young) children, using questions they are likely to understand. It has shown good criterion and construct validity in previous studies (Boyce et al., 2006).

#### Psychosocial Adjustment (Mental Health and Well-Being)

– KIDSCREEN-10 Index (Ravens-Sieberer and The KIDSCREEN Group Europe, 2006). This 10-item questionnaire assesses the subjective Health-Related Quality of Life (HRQL) and well-being in children and adolescents aged 8–18. For each item, five answer categories ranging from “never” to “always” or from “not at all” to “extremely” are provided. The 10 items of the KIDSCREEN-10 Index address affective symptoms of depressed mood: cognitive symptoms of disturbed concentration; psycho-vegetative aspects of vitality, energy, and feeling well; and psychosocial aspects correlated with mental health, such as the ability to experience fun with friends or getting along well at school. The index provides good discriminatory power, good internal consistency (Cronbach’s alpha = 0.82) and test–retest stability (*r* = 0.73; ICC = 0.72) (Aymerich et al., 2005).

#### Trait Emotional Intelligence

– Trait Emotional Intelligence Questionnaire-Child Short Form (TEIQue-CSF; Mavroveli et al., 2008), Spanish adaptation by Piqueras et al. (2017c). This questionnaire is designed to measure global TEI in children between 8 and 12; it includes 36 items, rated using a five-point Likert scale (1 = “strongly disagree” to 5 = “strongly agree”). The total score indicates global TEI. This questionnaire is available, free of charge for academic research purposes, from the London Psychometric Laboratory. In this sample, the internal consistency was 0.84.

#### Emotional Problems

– Revised Child Anxiety and Depression Scale, 30-item version (RCADS-30; Chorpita et al., 2000; Spanish version by Sandin et al., 2010). This is a reduced 30-item version of the RCADS, a self-report for evaluating anxiety and depression in children and adolescents. The scale comprises 30 items and 6 subscales for evaluating specific anxiety disorder symptoms; the present study has used the total score only. The scale ranges from 0 to 3 (corresponding to “never,” “sometimes,” “often,” and “always,” respectively). The scale has shown excellent psychometric properties, equivalent to the full version, with a Spanish population (α = 0.87) (Sandin et al., 2010; Piqueras et al., 2017b). For this sample, the alpha coefficients for the total score were 0.89.

#### Social Problems

– Sociometric Questionnaire for Children and Pre-adolescents (Cuestionario Sociométrico para Niños y Preadolescentes; CSN and CSP; Diaz-Aguado, 1995). This questionnaire is based on the “nominations method,” in which children are asked to name the three boys or girls in their group whom they are most likely to play with and to give reasons for their elections and rejections. They provide a rate of rejections and elections: between the number of possible rejections/elections and the total number of children.

## Design and Procedure

The sampling method was the intentional or convenience method. The inclusion criteria considered for the selection of schools were as follows: (1) to be a primary school located in the province of Alicante and (2) to have obtained authorization from the school management team to participate in the research. The inclusion criteria used to select children were: (1) to be in the second or third grade of primary education and (2) to have obtained the parents or the legal guardians’ signed informed-consent document that was approved by the Ethical Committee of University. Informed consent was not obtained from each individual participants included in the study because all of them were under 12 years of age, and Spanish legislation concerning the informed consent of the minor in health does require only the paternal approval and consent for this type of studies.

The children were told that the object of study was important and invited to participate on a voluntary basis; the confidentiality of their data was guaranteed. This approach obtained good participation in general. They were asked to provide socio-demographic data; the instructions for each of the battery tests were explained. The investigators who administered the tests responded to all questions and doubts that arose. The battery of tests took approximately 1 h to complete. Selected measures were applied through a web-based survey called DetectaWeb (further information in Piqueras et al., 2017a).

The design and execution of the research conformed to national and international ethical standards established for scientific research, receiving the approval of the Ethical Committee (Organo Evaluador de Proyectos) of the Miguel Hernandez University of Elche, reference number: DPS. JPR. 01.16.

## Statistical Analysis

**FIGURE 1:**
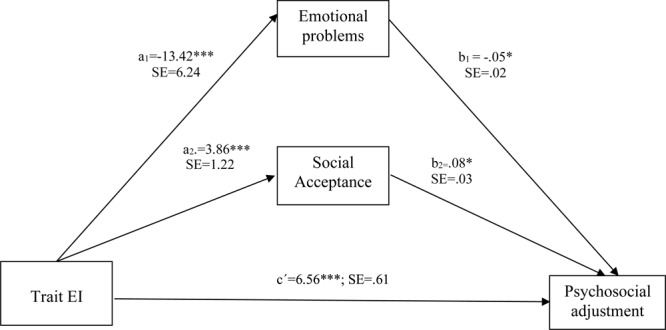
Model of the relationships among TEI, emotional problems, and acceptance as predictors and psychological adjustment as criterion. ^∗^*p* < 0.05, ^∗∗∗^*p* < 0.001.

First, preliminary analyses were carried out on the descriptive statistics. Secondly, analyses of the relationship between EI, emotional problems, and social acceptance and rejection were carried out using Pearson’s correlation coefficient, following Cohen (1988, 1992) recommendations to consider a correlation as indicative of a small (less than 0.29), medium (between 0.30 and 0.49) or large (0.50 or greater) effect size. Thirdly, to investigate the validity of EI, emotional problems, and acceptance and social rejection, as factors that could be used to determine psychosocial adjustment, we conducted four-step hierarchical regression analyses. We also conducted regression analyses to examine the role of emotional problems and acceptance and social rejection as mediators of the link between EI and psychosocial adjustment, based on the recommendations of Baron and Kenny (1986). A hierarchical regression analysis was conducted to examine a mediation/moderation model using the PROCESS tool (Hayes, 2013). To directly test our proposed mediation/moderation model (Figure 1), we used Model 14 in PROCESS to develop and analyze the role of gender in our previous mediational model.

## Results

### Descriptive Statistics and Correlations Between Key Variables

Descriptive statistics and inter-correlations for the study variables are shown in Table 1. Psychosocial adjustment was both positively and statistically related to TEI and social acceptance; it was negatively related to negative emotional symptoms and social rejection. TEI was both positively and statistically associated with social acceptance and negatively associated with negative emotional symptoms and social rejection. Finally, negative emotional symptoms were positively and statistically associated with social rejection.

**Table 1:** Descriptive statistics and inter-correlations among the measures.

	Mean	*SD*	1	2	3	4	5
(1) TEI	3.71	0.49	–				
(2) Emotional symptoms	28.29	13.66	–0.507^∗∗^	–			
(3) Social acceptance	9.91	7.98	0.227^∗∗^	–0.097	–		
(4) Social rejection	5.78	8.83	–0.163^∗∗^	0.207^∗∗^	–0.333^∗∗^	–	
(5) Psychosocial adjustment	41.65	5.73	0.647^∗∗^	–0.420^∗∗^	0.292^∗∗^	–0.174^∗∗^	–

*TEI, Trait Emotional Intelligence; ^∗∗^p < 0.01.*

We found a large effect between TEI and Emotional Problems and between TEI and Psychosocial Adjustment; there was a moderate effect between social acceptance and rejection, emotional problems and psychosocial adjustment, and peer acceptance and psychosocial adjustment.

### Analyses of Hierarchical Regression

To examine the validity of TEI, negative emotional symptoms, and acceptance and rejection as determinants of psychosocial adjustment, we conducted a four-step hierarchical regression (see Table 2).

**Table 2:** Analyses of hierarchical regression.

	*B*	*SE*	*B*	*R*^2^	Δ*R*^*2*^	*F*(df)
**Step 1**				0.003	–0.001	0.693 (1,265)
Gender	0.587	0.705	0.051			
**Step 2**				0.436**	0.432**	102.177^∗∗∗^ (2,264)
Gender	1.354	0.534	0.118			
TEI	7.726	0.542	0.662***			
**Step 3**				0.450**	0.444**	71.850^∗∗∗^ (3,263)
Gender	1.182	0.532	0.103			
TEI	6.964	0.611	0.597***			
Emotional symptoms	–0.057	0.022	–0.136***			
**Step 4**				0.473**	0.463**	46.827^∗∗∗^ (5,261)
Gender	1.115	0.524	0.097			
TEI	6.561	0.614	0.562***			
Emotional symptoms	–0.055	0.022	–0.132**			
Social acceptance	0.088	0.035	0.123**			
Social rejection	–0.040	0.031	–0.061			

*TEI, Trait Emotional Intelligence; ^∗∗^p < 0.01, ^∗∗∗^p < 0.001.*

The determinant variables were gender, TEI, negative emotional symptoms, and acceptance and rejection. The dependent variable was psychosocial adjustment. We conducted the regression by first entering gender into the model, followed by TEI and negative emotional symptoms, and finally by acceptance and rejection.

The results of the regression model are shown in Table 2. Gender was not a significant determinant of psychosocial adjustment. TEI, added to the models during the second step, proved to be a significant determinant of psychosocial adjustment (Δ*R*^2^ = 0.432), with higher scores in TEI determining higher psychosocial adjustment. Negative emotional symptoms proved to be a significant determinant of psychosocial adjustment (Δ*R*^2^ = 0.444). The last step in the model included acceptance and rejection, but just the former proved to be a significant determinant of psychosocial adjustment (Δ*R*^2^ = 0.463).

### Mediation/Moderation Analyses

Previous analyses of regression have shown that when TEI, negative emotional symptoms, and social acceptance and rejection are simultaneously included in the model, these variables account for 46% of the variance in psychosocial adjustment, when gender has been controlled for (see Table 2).

This result suggests that both constructs are relevant in determining the psychosocial adjustment of children; to assess this idea, we constructed a mediation/moderation model to test the relationship between these variables. First, we investigated the hypothesis that negative emotional symptoms and social acceptance and rejection can be an important mechanism in the relationship between TEI and psychosocial adjustment in children.

In particular, we tested whether negative emotional symptoms and acceptance and social rejection mediate the relationship between TEI and psychosocial adjustment (see Table 3).

**Table 3:** Summary of multiple mediation/moderation analyses of trait emotional intelligence and psychosocial adjustment (5,000 bootstrap samples).

Independent variable (IV)	Mediators	Dependent variable (DV)	Effect of IV on M (a)	Effect of M on DV (b)	Total effect (c)	Direct effect (c′)	Indirect effect (c–c′)	Effect on DV through proposed mediators covariates	(CI) 95%
TEI		Psychosocial adjustment			7.72^∗^	6.56^∗∗∗^	1.16^∗^	Gender: 1.11^∗^	
	Emotional problems		–13.42^∗∗∗^	–0.05^∗^			0.74^∗^		0.1481 to 0.14973
	Social acceptance		3.86^∗∗∗^	0.08^∗^			–0.34^∗^		0.0965 to 0.7330
	Social rejection		–0.26	–0.04			0.01		–0.0818 to 0.2257

*^∗^p < 0.05, ^∗∗∗^p < 0.001.*

As can be seen in Route C, Figure 1, this mediation/moderation analysis showed that children with higher TEI had better psychosocial adjustment. In addition, Route A, in Figure 1 shows that TEI was negatively associated with emotional problems and positively associated with social acceptance, suggesting that children with a higher level of TEI have fewer emotional problems and higher levels of social acceptance. Finally, as can be seen in Route B in Figure 1, emotional problems were negatively related to psychosocial adjustment, while social acceptance was positively related to psychosocial adjustment.

Using an indirect procedure, 95% CI bootstrapped confirmed that TEI exerted an indirect, positive, and significant effect on psychosocial adjustment through emotional problems and social acceptance. This result suggests that emotional problems and social acceptance partially mediate the association between TEI and psychosocial adjustment. Since previous research indicates that emotional problems, social acceptance, and rejection may be influenced by gender, with girls reporting higher scores for emotional problems (García-Olcina et al., 2014; Del Barrio and Carrasco, 2016), this study tested the role of gender as a possible covariate of the observed robust link between TEI and psychosocial adjustment (Table 3), which was significant.

## Discussion

The aim of this study was to examine how TEI, emotional problems, and social acceptance and rejection affected the psychosocial adjustment of children. As a further step, this study examined whether emotional problems, social acceptance, and rejection play a role in mediating the relationship between TEI and psychosocial adjustment, and whether gender is a moderating variable in this relationship.

Multiple regression and mediation/moderation analyses revealed the way in which psychosocial adjustment in children was determined by TEI, emotional problems, and social acceptance. In particular, higher scores in TEI and social acceptance seemed to predict better psychosocial adjustment. Likewise, a higher score on emotional problems seemed to predict a worse psychosocial adjustment. These findings are consistent with our first hypothesis; although relatively few studies have evaluated the specific relationship between EI and psychosocial adjustment in children, the results of a study with adolescents reveal a relationship between higher levels of TEI and better psychosocial adjustment (Palomera et al., 2012). Another study carried out on the adolescent and adult population has revealed that high TEI is associated with the absence of social stress, anxiety, and depression (Barraca and Fernández, 2006). Mateu-Martínez (2017) studied children between the ages of 8 and 12 and found that social acceptance was more closely related to TEI, and less closely related to the symptomatology of emotional problems, such as anxiety and depression. By contrast, social rejection is related to emotional problems that can be considered indicators of imbalance, such as symptoms of social phobia, depression, and low self-esteem. Nonetheless, social rejection was not significant in our model as a predictor of psychosocial adjustment in the presence of TEI, emotional problems and social acceptance as competing predictors.

Interestingly, the present study found that emotional problems and social acceptance play a role in moderating, at least in part, the association between TEI and psychosocial adjustment. Higher TEI levels in children are therefore related to higher levels of psychosocial adjustment through lower emotional problem scores, a finding consistent with our second hypothesis.

Emotional problems have been shown to be a moderating variable in a wide range of outcomes (Aluja and Blanch, 2004; Park et al., 2010; Reinke et al., 2012; Bernaras et al., 2013). However, in our opinion, this is the first study to look at this moderation effect in children. The present study has found that emotional problems partially mediate the relationship between TEI and psychosocial adjustment in children. In terms of educational and clinical implications, this moderation effect is of critical importance, since it opens the door to new lines of intervention that focus on improving emotional problems in the educational and clinical settings of these populations.

In educational and clinical settings, the ultimate goal of emotional education interventions is to promote psychosocial adjustment and well-being of children. Emotional education programs in primary school students provoked a significant improvement in emotional competences, social relationships, and psychosocial adjustment (i.e., Filella-Guiu et al., 2014; Cejudo, 2017). Our results point to the need to consider the presence of emotional and social problems in such interventions. Therefore, any intervention should evaluate these dimensions, as well as include, among the objectives of the programs, the development of emotional and social competences to overcome these difficulties.

Similarly, social acceptance plays a role in moderating the partnership between TEI and psychosocial adjustment. Therefore, higher TEI levels in children are related to higher levels of psychosocial adjustment, through higher social acceptance scores, a finding that is consistent with our second hypothesis and other studies with children (Petrides et al., 2006; Mavroveli et al., 2008; Agnoli et al., 2012; Andrei et al., 2015; Banjac et al., 2016) and with adolescents (Povedano et al., 2012; Mancini et al., 2017) in which children and adolescents involved in rejection situations showed less adaptive psychosocial profiles and those most vulnerable to social rejection are those who do not succeed in developing adequate levels of TEI, presenting more difficulties.

Future research should focus on whether EI programs for children could reduce the children’s symptoms of emotional problems and improve their relationships with peers, benefiting both present and future child psychosocial adjustment. There is already some evidence regarding the efficacy of this type of intervention, for example, in the Mateu-Martínez (2017), which observed improvements in psychological strength variables related to both short-term and long-term emotional adjustment. The adjustment reduced symptoms of emotional problems, suggesting that this may be a promising field for future interventions with children.

Similarly, future research should also focus on seeking additional mediators between TEI and psychosocial adjustment during childhood. For example, some indicators of school adjustment are academic achievement and classroom behavior. In perceptual attributes associated with social acceptance and rejection, children guided their rejection of behaviors that annoyed others to lower intellectual capacity, basing their choices on aspects related to helping behaviors, or to considering a partner more intelligent, which can be a protective factor for children in the academic environment (Mateu-Martínez, 2017).

In the present study, gender emerged as a moderator of the link between TEI and psychosocial adjustment, which is consistent with our third hypothesis. Specifically, for the girls in this study, lower TEI scores determined lower levels of psychosocial adjustment, regardless of emotional problems or social acceptance and rejection. These results are consistent with those previously reported in the literature, along with the assertion that gender is one of the individual factors that can predict differences in psychosocial adjustment (Palomera et al., 2012). Gender differences also suggest potentially novel areas of research, as there is still no clear consensus on the association between gender-specific relationships and psychosocial adjustment. Generally, girls receive a more emotion-focused education, while boys are taught to reduce certain emotions (Fivush et al., 2000; Sánchez-Núñez et al., 2008).

Of paramount importance is to emphasize that our non-clinical sample was composed of children, a population where there have been considerably fewer studies and where prevention is easier than in older individuals. In fact, childhood is an ideal time to train and modify undesirable behavior patterns. Despite our findings, we are aware of the desirability of replicating these studies with a larger sample and wider range of ages, to enable these results to be widely generalized.

On the other hand, the exclusive use of self-reporting measures may be another limitation in terms of assessment, as social desirability and premeditated bias may affect responses to the questionnaires of social and emotional problems. Anyway, it should be noted this is not the case for the assessment of TEI, given its nature as a combination of self-efficacy and affective-personality dispositions requires the use of self-reports (see Pérez et al., 2005). Despite the various potential sources of error in this type of instrument, compared to other standardized techniques, self-reports remain valid and reliable measures. As different authors, such as Lundqvist et al. (2010) or Górriz et al. (2015), have pointed out, they are the best source of information when assessing internalized problems. Participants find it more convenient to explore their own thoughts and feelings, given that they have direct knowledge of their own inner states. Their answers are considered reliable from the age of eight onwards (Lundqvist et al., 2010; Górriz et al., 2015). Future researchers are therefore advised to replicate this research structure, including assessment measures completed by different informants, such as families and teachers, in order to achieve a better understanding of the problem and its scope, covering so the identity and reputation components of personality (e.g., Hogan, 1983).

Following the proposal made by Martins et al. (2010), based on the model of this investigation, the present study used the short version for children of the TEIQue (Mavroveli et al., 2008), one of the most widely used measures of TEI (Di Fabio and Kenny, 2016). However, in studies conducted using the adult version of this instrument, the TEIQue appears to be more related to intrapersonal EI aspects and well-being, and to a lesser extent interpersonal EI; it would therefore be useful for EI instruments to explore both aspects equally.

In line with the limitations found in similar studies, it would be advisable to design longitudinal studies using children and adolescents between 8 and 18 years of age, from different regions of the same country. Likewise, we studied concurrent relationships between predictors and criteria, given both were assessed in the same time. We also recommend using a combined variety of assessment measures, including self-reports and others-reports, which have high reliability and validity, in addition to objective measures. It may be useful to add new variables that are significant to child psychosocial adjustment, such as family and school adjustment. These could include academic performance or classroom behavior (Filella-Guiu et al., 2014; Cejudo, 2017; Pérez-González and Qualter, 2018).

## Conclusion

Our results replicate in children the positive association between TEI and well-being previously observed in young people and adults, over and above the predictive power of social and emotional problems (e.g., Martins et al., 2010; Petrides et al., 2016, 2017). In particular, this study empirically supports the interpretation of TEI as a buffer of stressful circumstances for psychosocial adjustment in children, what could constitute an advantage for the later development in youth and adults. One of the main contributions of this study is that it focuses on child population, since most previous research has been carried out on adults or adolescents, bypassing the study of childhood. Research carried out on EI and psychosocial adjustment in children is still incipient (Mavroveli et al., 2008), despite the personal and social consequences of a deficit development in certain areas (emotional, cognitive, and social) or the problems of imbalance at this evolutionary stage (Lahaye et al., 2013; Eastabrook et al., 2014; Rowsell et al., 2014).

These research findings show that TEI and emotional problems and social acceptance are both determinants of psychosocial adjustment in children aged 8–12, supporting the idea that these factors are fundamental to understanding psychosocial adjustment. In addition, emotional problems and social acceptance play a role in moderating the relationship between TEI and psychosocial adjustment, mainly for girls in the present sample. This finding is consistent with Andrei et al. (2015)’s study, who found that gender moderates the association of TEI with actual social status, predicting TEI perceived social acceptance only for female children and adolescent (Andrei et al., 2015). Future studies are needed to replicate these findings and to further explore the effects of gender on TEI linkages, emotional problems, social acceptance and rejection, and psychosocial adjustment.

Finally, this study provides a new vision for understanding the effects of TEI and socio-emotional problems on child psychosocial adjustment. From an applied perspective, research into and knowledge of the factors that influence TEI can help to develop tools and interventions to promote positive aspects and to help children adapt during times of conflict or evolutionary transitions (Goldner and Scharf, 2013). More specifically, their relationship with different emotional and social variables can mediate psychosocial adjustment. In this sense, it would be interesting to design emotional education programs to promote TEI of children, including those variables that research has identified as most relevant, in order to prevent problems of maladjustment in childhood. Concerning the gender differences observed in the mediation/moderation analyses, lower TEI scores determined lower levels of psychosocial adjustment specifically in girls. Given this finding, our study suggests that girls in age group 8–12 could constitute a preferential prevention group in emotional education because of their greater potential vulnerability to the effects of low levels of emotional competence.

## Author Contributions

OM-M conceived of the study, participated in the data collection, led the preparations, and wrote the first draft of the manuscript. JC and J-CP-G analyzed the data and wrote the first draft of the manuscript. JP conceived of the study, analyzed the data, and contributed to writing the manuscript. All authors contributed to interpreting the data, helped to draft and revise the manuscript, and read and approved the final manuscript.

## Conflict of Interest Statement

The authors declare that the research was conducted in the absence of any commercial or financial relationships that could be construed as a potential conflict of interest.
